# Carbon quantum dots with honeycomb structure: a novel synthesis approach utilizing cigarette smoke precursors

**DOI:** 10.1038/s41598-024-52106-3

**Published:** 2024-01-23

**Authors:** Setianto Setianto, Liu Kin Men, Ayi Bahtiar, Camellia Panatarani, I Made Joni

**Affiliations:** 1 Department of Physics, FMIPA, Padjadjaran University, Jl. Raya Bandung-Sumedang KM 21, Sumedang, 45363 Jawa Barat Indonesia; 2 Functional Nano Powder University Centers of Excellence, Padjadjaran University, Jl. Raya Bandung-Sumedang KM 21, Jatinangor, 45363 Jawa Barat Indonesia

**Keywords:** Green chemistry, Materials chemistry, Graphene, Optical properties and devices

## Abstract

This study presents a novel approach to synthesizing honeycomb carbon quantum dots (CQDs) from cigarette smoke by a hydrothermal process. A comprehensive characterization of these CQDs, conducted through high-resolution transmission electron microscopy (HRTEM), showcases their unique honeycomb structure, with an average particle size of 6.3 nm. Photoluminescence (PL) in CQDs is a captivating phenomenon where these nanoscale carbon structures emit strong blue luminescence at 461 nm upon exposure to ultraviolet light, with their excitation peak occurring at 380 nm. Fourier Transform Infrared (FTIR) analysis also identifies specific functional groups within the CQDs, offering valuable insights into the mechanisms governing their photoluminescence. Analysis of excitation spectra indicates the presence of both aromatic C=C bonds at 254 nm and C–O bonds from 280 to 420 nm.

## Introduction

Carbon Quantum Dots (CQDs) have emerged as a fascinating class of nanomaterials due to their unique optical properties and potential applications in various fields, including optoelectronics, bio-imaging, sensing^1,2^, and energy conversion^3–9^. They are nanoscale carbon-based particles with sizes typically less than 10 nm, consisting of a graphitic core surrounded by a surface passivated with functional groups^10,11^. Quantum confinement effects influence the size-dependent photoluminescence of CQDs, making them extremely appealing for fluorescent labeling and imaging applications^12^. Previous researchers have utilized various precursors and synthesis methods in the synthesis of CQDs. They have employed precursors such as citric acid, sucrose, lignin, and biomass waste like agricultural residues^11,12^. These precursors were then processed using different synthesis methods, including hydrothermal methods under high temperature and pressure conditions, pyrolysis methods involving high-temperature heating in an inert atmosphere^15^, electrochemical methods involving the application of an electric potential or current to a precursor solution containing carbon sources^16^, and laser ablation methods utilizing laser beams to convert carbon precursors into CQDs^17,18^. Therefore, ongoing research aims to explore alternative precursors and synthetic methods to improve the potency and optoelectronic properties of the resulting CQDs. Thus, using waste materials as precursors in CQD synthesis has become a sustainable and cost-effective method that contributes to environmental protection and economic efficiency^19–21^. By reusing waste materials that would otherwise be, this approach reduces waste encourages recycling, and provides an affordable alternative to traditional synthesis methods. Using scrap materials for CQD synthesis not only reduces waste and promotes recycling, but also provides a cost-effective replacement for traditional synthesis techniques. By using waste materials, the costs associated with the production of CQDs can be minimized. By implementing this strategy, the conversion of cigarette smoke into valuable nanomaterials is in line with the principles of green chemistry and the circular economy, paving the way to a more sustainable future^22,23^. The hydrothermal technique, recognized for its simplicity and effectiveness, offers a precise approach to synthesizing CQDs with customizable properties. Within the hydrothermal environment, researchers can control the growth of CQDs, allowing fine-tuning of various factors such as temperature, pressure, and reaction time^18,24^. In a significant departure from conventional approaches, our research pioneers a transformative method that harnesses cigarette smoke, a notorious environmental pollutant, and converts it into honeycomb-like Carbon Quantum Dots (CQDs) using a green and chemical-free process. This innovation not only addresses the urgent concern of cigarette smoke pollution but also yields CQDs with tailored properties, unlocking exciting possibilities. Furthermore, our investigation delves into the specific optical properties of these CQDs, unraveling their precise electronic transitions. This groundbreaking approach, blending remediation efforts with nanomaterial synthesis, presents a targeted solution to the challenges associated with cigarette smoke pollution.

## Experimental methods

### Synthesis of carbon quantum dots

**Figure 1:**
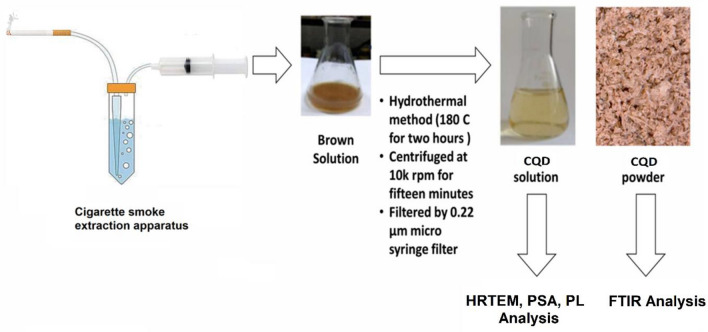
The procedure for synthesizing Carbon Quantum Dots (CQDs), provides a visual representation of the synthesis process.

Figure 1 provides a visual representation of the synthesis process for carbon quantum dots (CQDs), illustrating a detailed explanation of the sequential steps involved in synthesizing CQDs.

CQD precursors are made through a cigarette smoke extraction process which involves collecting and condensing cigarette smoke particles into water with simple equipment following the protocol carried out by Gellner et al.^25^, which is then allowed to stand for ten weeks. The water will turn brown after this duration. These labeled as CSW (Cigarette Smoke Water extract), were further processed using a hydrothermal method. A 50 mL portion of CSW was transferred to a stainless steel autoclave, which was sealed and heated to 180 °C at a ramp rate of 5 °C min^−1^. The autoclave was maintained at 180 °C for 2 h to facilitate the carbonization process. Afterward, the autoclave was cooled down to room temperature at a ramp rate of 5 °C min^−1^. The resulting dark brown material obtained after cooling indicated the formation of carbonaceous structures derived from cigarette smoke. The carbonized product was isolated and purified through dissolution in deionized water, subsequent filtration using a 0.22-micron Millipore syringe filter, and centrifugation to eliminate larger particles and impurities. The supernatant containing the carbon quantum dots was collected, and after solvent evaporation, the CQDs were obtained as a powder suitable for further analysis and characterization*.*

### Characterization of carbon quantum dots

"In the experimental phase, we conducted Fourier Transform Infrared (FTIR) analysis to identify the presence of functional groups in the synthesized CQDs. This analytical technique allowed us to characterize the molecular composition and verify the formation of specific functional groups within the CQDs." FTIR of CQDs was performed on a Nicolet iS50 spectrometer (Thermo Fisher Scientific, USA). "Additionally, High-Resolution Transmission Electron Microscopy (HRTEM) analysis was employed to gain critical insights into the morphology and microstructure characteristics of the carbon quantum dots. This technique provided detailed information about the size, shape, and structural features of the synthesized CQDs." The specific HRTEM instrument used in the study was a JEOL JEMCXII, operating at an acceleration voltage of 200 kV. Carbon quantum dot samples were prepared by depositing a small amount of the synthesized material onto a suitable substrate, such as a carbon-coated copper grid. Images and electron diffraction patterns obtained from HRTEM provided valuable insights into the crystal structure, lattice spacing, and overall morphology of the carbon quantum dots. The Horiba SZ-100 instrument employs dynamic light scattering (DLS) and electrophoretic light scattering (ELS) methods to examine the distribution of particle sizes and the electric potential difference between the surface of particles and the liquid surrounding them. This instrument is specifically utilized for analyzing the size distribution and the surface charge characteristics of carbon quantum dots (CQDs). The photoluminescence properties of the carbon quantum dots in an aqueous solution were analyzed using a photoluminescence setup. The specific photoluminescence setup used in the study was a Perkin Elmer LS 55, provided by Perkin Elmer Singapore PTE Ltd. The photoluminescence spectra were recorded by exciting the carbon quantum dots with a specific wavelength of light and measuring the emitted fluorescence.

## Results and discussion

### Structural characterization of carbon quantum dots

**Figure 2:**
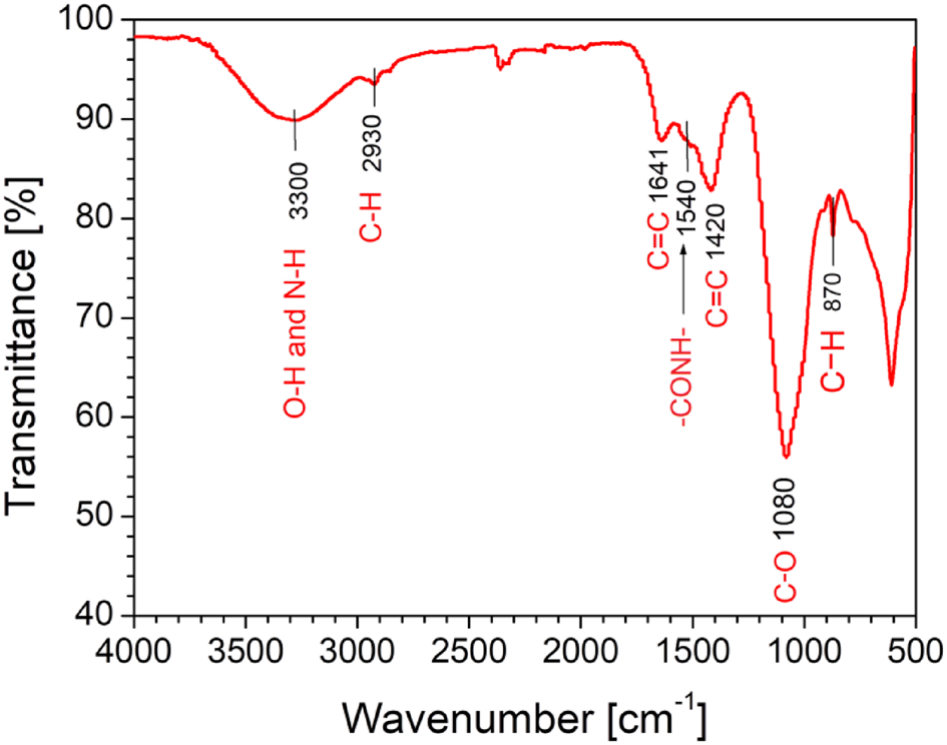
FTIR spectroscopy of synthesized Carbon Quantum Dots powder.

In the subsequent discussion of our findings, the results from the FTIR analysis confirmed the presence of distinct functional groups within the carbon quantum dots, contributing to our understanding of their chemical composition. This information is pivotal for elucidating the surface properties and potential applications of the CQDs. Figure 2 displays the FTIR spectrum of the carbon quantum dots, where various functional groups exhibit distinct peaks or bands at specific wavenumbers. The vibrations at 3300 cm^−1^ and 2930 cm^−1^ indicate the presence of N—H/O—H and C—H bonds, respectively, suggesting the existence of amines or hydroxyl groups and methylidene groups within the CQDs. The vibration at 1641 cm^−1^ and 1420 cm^−1^ corresponds to aromatic C=C bonds, indicating the presence of aromatic ring systems within the CQDs. The vibration at 1540 cm^−1^ signifies the presence of the –CONH- bond, indicating the possibility of amides. The vibrations at 1080 cm^−1^ and 870 cm^−1^ indicate the presence of C-O bonds and various types of C-H bonds, respectively, providing insights into the presence of oxygen-containing functional groups and different carbon-hydrogen moieties in the CQDs. The FTIR spectrum analysis of CQDs in this study provides valuable insights into their properties and potential applications. The presence of functional groups, such as N–H, O–H, and C-O bonds, indicated by peaks at 3300 cm^−1^ and 1080 cm^−1^, enhances the surface reactivity of CQDs, making them suitable for diverse applications. The presence of C-H bonds, signified by the peak at 2930 cm^−1^, confirms the carbon-based structure of CQDs, enhancing their stability and compatibility with materials like graphene. The peak at 1540 cm^−1^ indicates the presence of amide groups (–CONH– bond) which contribute to the CQDs' surface reactivity. The peaks at 1641 cm^−1^ and 1420 cm^−1^ suggest the presence of aromatic C=C bonds in the ring system, which enhances the optical properties, stability, and conductivity of CQDs.

### Morphology of carbon quantum dots

**Figure 3:**
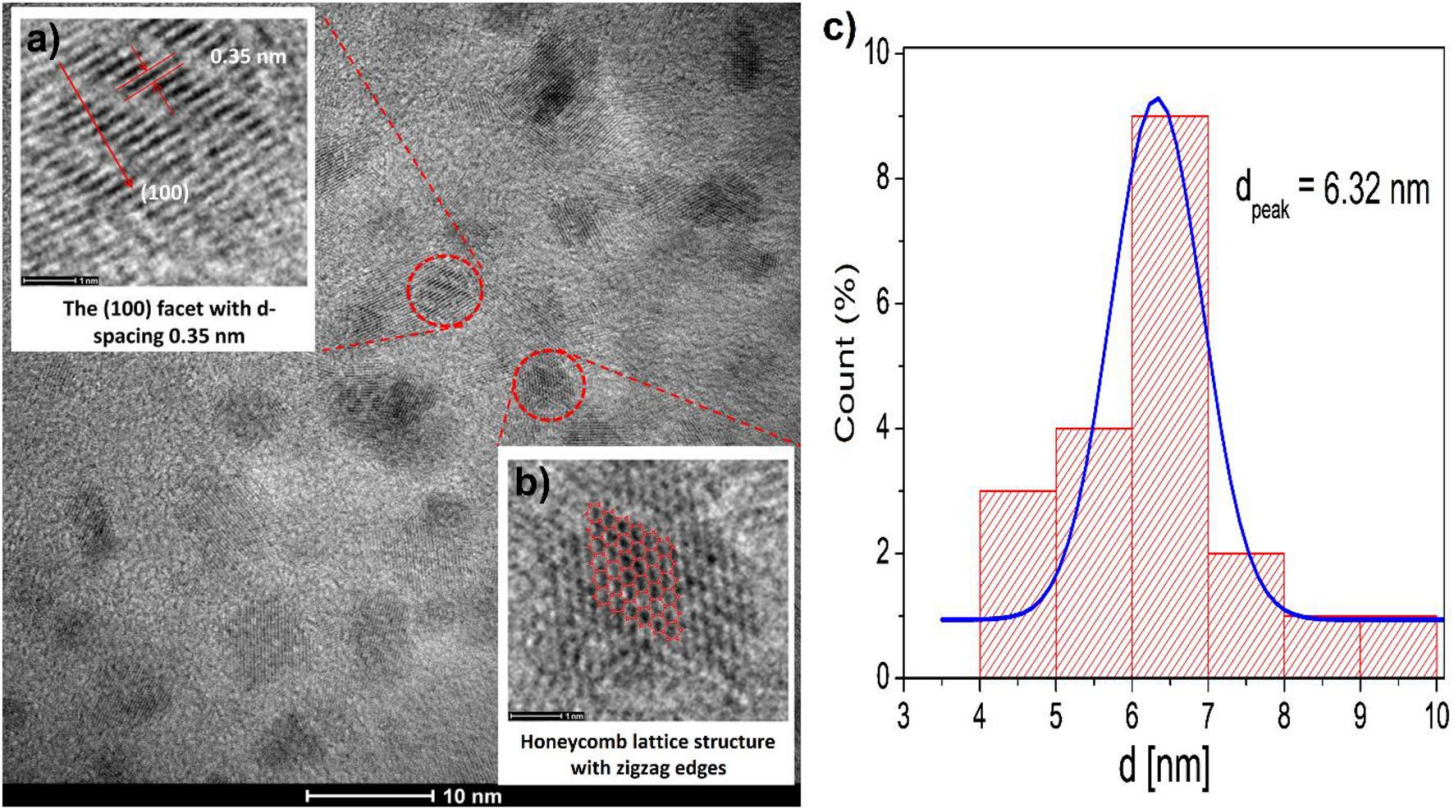
High-resolution transmission electron microscopy (HRTEM) image and particle size distribution histograms of the carbon quantum dots. Additionally, the HRTEM analysis reveals a honeycomb lattice structure within the CQDs, as indicated by a measured the (100) facet with a layered spacing of 0.35 nm and including the determined particle size of CQDs is 6.32 nm.

The HRTEM analysis unveiled crucial details about the morphology and microstructure characteristics of the carbon quantum dots. The observed size, shape, and structural features provide essential insights that are instrumental in comprehending the unique properties of the synthesized CQDs. These findings open avenues for tailored applications and highlight the significance of the CQD structure in influencing their properties. Figure 3 shows the morphology and nanostructure of the CQD sample obtained from HRTEM observation. Figure 3a displays the HRTEM analysis results, indicating that the CQD particles possess a nearly spherical shape, characterized by a rounded and symmetrical appearance. The nearly spherical shape of the CQD particles suggests that they possess structural stability, size uniformity, an enhanced surface-to-volume ratio, and the capability to disperse effectively in different solutions. Additionally, this morphology offers the potential for distinctive optical properties. The close-to-spherical structure is advantageous as it enhances the stability of the CQDs, their dispersibility in diverse media, and their ability to interact with other substances or environments^26–28^. A significant discovery was the identification of a honeycomb structure within the CQDs, exhibiting a pattern reminiscent of the hexagonal lattice observed in graphene and carbon nanotubes (Fig. 3b). This honeycomb structure offers several advantageous properties such as structural stability, excellent electrical conductivity, and a large surface area, which are highly desirable for various applications^26,29,30^. Additionally, the HRTEM analysis revealed that the CQDs have a rhombus shape, indicating that they possess four sides of equal length with parallel opposite sides. This unique shape adds to their structural diversity and may impact their optical and surface properties^31^. Moreover, the analysis demonstrated that the lattice spacing within the honeycomb structure of the CQDs measures 0.24 nm. This lattice spacing indicates the nanoscale size and arrangement of carbon atoms within the CQDs^32–34^. In Fig. 3c, particle size distribution and particle diameter (d_peak_) were evaluated from HRTEM images, revealing that smaller particles measure approximately 6.32 nm.

### Particle size analysis of carbon quantum dots

**Figure 4:**
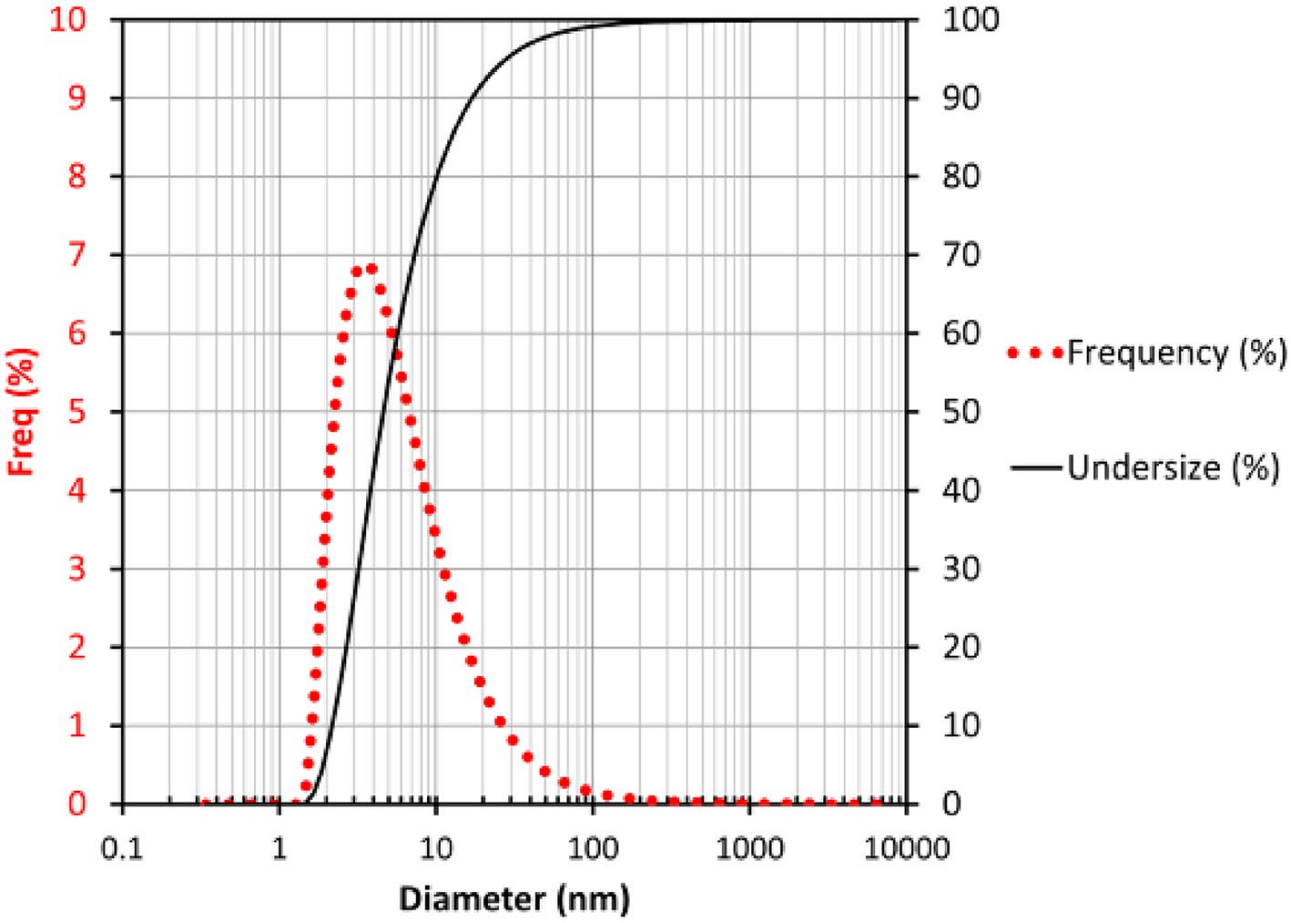
The particle size distribution of the synthesized carbon quantum dots (CQDs).

Particle Size Analyzer (PSA) analysis is commonly employed to determine the size distribution of particles in a solution and characterize the size of carbon quantum dots. Through PSA analysis, the average size of carbon quantum dots is calculated, representing the central tendency or mean size of the particle population. In Fig. 4, the average size of carbon quantum dots was measured to be 6.3 nm. Additionally, the carbon quantum dots exhibit a broad size distribution, indicating variations in size and revealing the heterogeneous nature of the sample. This characteristic offers valuable insights into the diversity and non-uniformity of the carbon quantum dots present in the sample^32,35,36^.

### Zeta potential of carbon quantum dots

**Figure 5:**
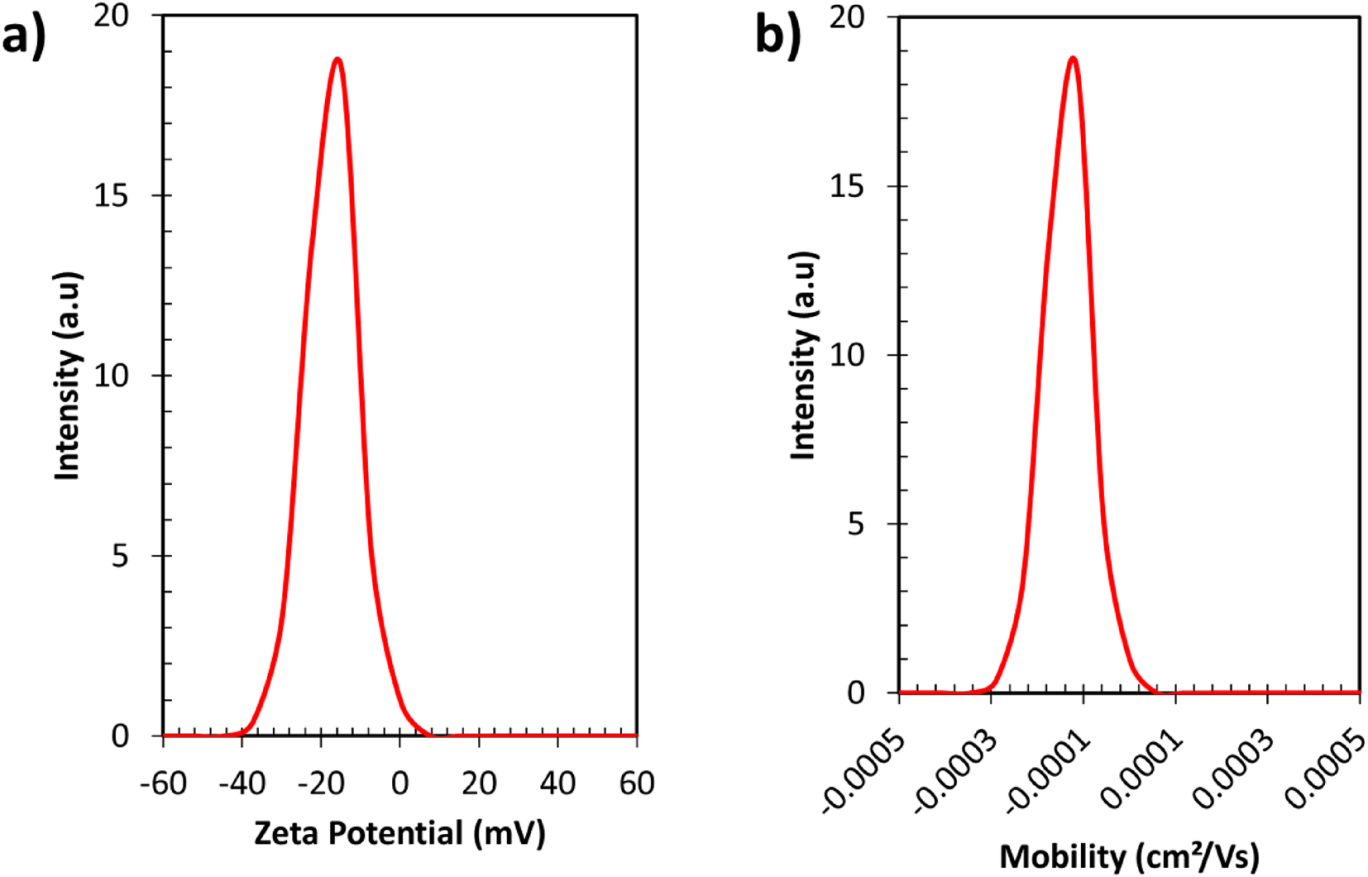
(**a**) The Zeta potential and (**b**) the electrophoretic mobility of Carbon Quantum Dots.

The Zeta potential and electrophoretic mobility are important parameters used to characterize the surface charge and movement of Carbon Quantum Dots (CQDs) in a solution. The Zeta potential represents the net electrical charge on the surface of the CQDs. In this case, the Zeta potential of −17.5 mV indicates that the CQDs have a negative surface charge as given in Fig. 5.

This negative charge arises from the presence of functional groups or chemical species on the surface of the CQDs that ionize in the solution, resulting in an accumulation of negatively charged particles around the CQDs. Electrophoretic mobility is a crucial parameter that measures the speed at which charged particles, in this case, carbon quantum dots (CQDs), move through a solution in response to an applied electric field. In this case, the electrophoretic mobility mean is −0.000135 cm^2^/Vs. Since the magnitude is relatively small (close to zero), it implies that the CQDs move slowly under the influence of the electric field. The presence of surface functional groups on carbon quantum dots (CQDs) is responsible for their negative charge and the migration towards the positive electrode in an electric field, resulting in negative values for both zeta potential and electrophoretic mobility. These surface functional groups typically consist of anionic chemical species or groups that contribute to the overall negative charge of the CQDs such as Carboxyl (–COOH), Hydroxyl (-OH), and Amino (–NH_2_).

### Photoluminescence spectra (PL)

**Figure 6:**
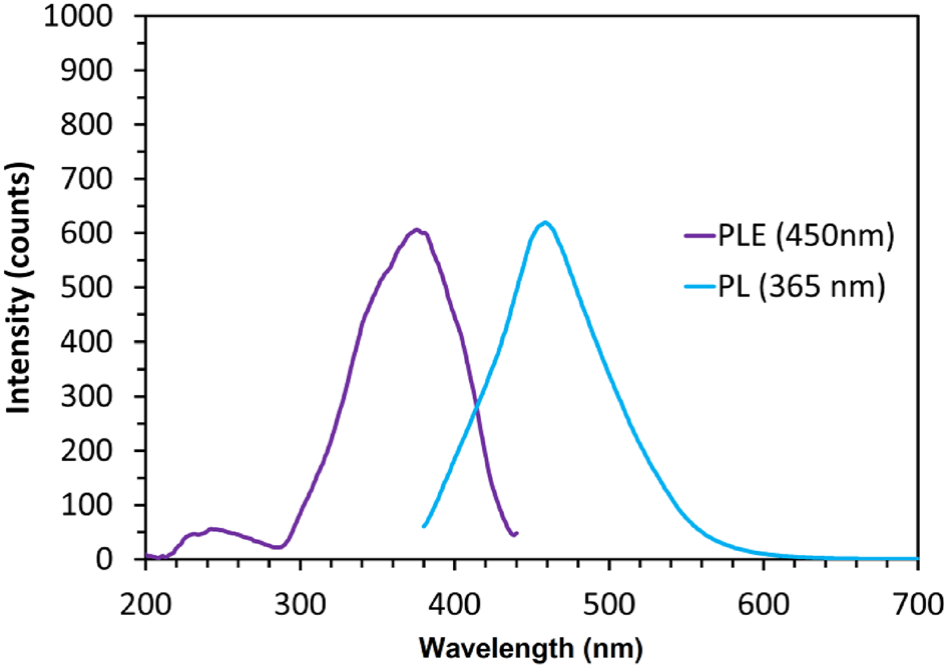
The photoluminescence (cyan line) and photoluminescence excitation (violet line) spectra of the synthesized carbon quantum dots.

Figure 6 displays the photoluminescence (PL) and photoluminescence excitation (PLE) spectra of the synthesized carbon quantum dots. The PL spectrum illustrates the emitted light when the CQDs are excited, providing insights into their optical properties. In this study, it was observed that the carbon quantum dots exhibited strong blue luminescence at a wavelength of 461 nm.

In contrast, the PLE spectrum demonstrates the absorbed light revealing the energy levels at which the CQDs can absorb photons and transition to higher energy states. In this scenario, the CQDs sample exhibits an absorption peak at 380 nm, leading to the emission of light at 450 nm when stimulated.

**Figure 7:**
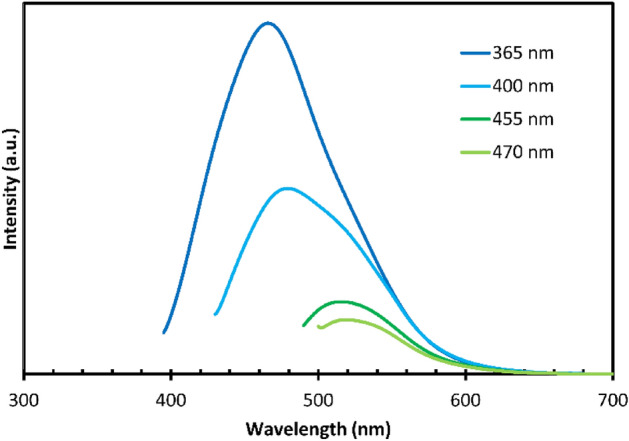
Excitation-dependent photoluminescence spectra of CQDs.

In Fig. 7, the excitation-dependent photoluminescence spectra of a CQD sample are illustrated, accompanied by the corresponding colors observed under different excitation conditions. This analysis provides a comprehensive understanding of how the CQDs respond to varying excitation wavelengths.

### Electronic transition mechanism

The FTIR analysis of the carbon quantum dots sample provides valuable insights into the PLE process, shedding light on the electronic transitions and molecular properties that contribute to the observed excitation peaks. By correlating the FTIR data with the PLE measurements, we gain a deeper understanding of the involvement of specific functional groups, such as aromatic C=C bonds and C-O bonds, in the electronic transitions and luminescence mechanism of the CQDs. The π → π* transition of aromatic C=C bonds, observed as a small excitation peak at 244 nm, involves the excitation of π electrons from the valence band to higher energy π* antibonding orbitals. Additionally, the stronger excitation peak in the range of 300 nm to 420 nm corresponds to the n → π* transition of C-O bonds, where non-bonding (n) electrons are excited to the π* antibonding orbitals^31^. These electronic transitions play a crucial role in the absorption and emission processes, with the aromatic C=C and C–O bonds contributing to the photoluminescence behavior of the CQDs^37,38^.

## Conclusion

In conclusion, this study successfully employed an eco-friendly hydrothermal method to transform cigarette smoke into honeycomb-structured carbon quantum dots (CQDs). A range of analytical techniques was applied to characterize the CQDs, revealing distinctive functional groups and confirming their unique morphology. Photoluminescence excitation measurements provided insights into the electronic transitions within the CQDs, and high-resolution transmission electron microscopy visually confirmed the honeycomb structure. These findings underscore the viability of using cigarette smoke as a source for CQD synthesis, opening new avenues for resource utilization. Moreover, the study's exploration of the optical properties of the CQDs enhances their potential applications in diverse fields, such as optics and electronics.

## Data Availability

All data used for this study are contained in this article.
